# Perinatal Origins of Adult Disease and Opportunities for Health Promotion: A Narrative Review

**DOI:** 10.3390/jpm12020157

**Published:** 2022-01-25

**Authors:** Stefano Nobile, Chiara Di Sipio Morgia, Giovanni Vento

**Affiliations:** Department of Woman, Child and Public Health, Division of Neonatology, Fondazione Policlinico Universitario Agostino Gemelli IRCCS, Università Cattolica del Sacro Cuore, 00168 Rome, Italy; chiara.dsm93@gmail.com (C.D.S.M.); giovanni.vento@unicatt.it (G.V.)

**Keywords:** disease, origin, development, developmental programming, perinatal, health

## Abstract

The “developmental origins of health and disease” (DOHaD) hypothesis refers to the influence of early developmental exposures and fetal growth on the risk of chronic diseases in later periods. During fetal and early postnatal life, cell differentiation and tissue formation are influenced by several factors. The interaction between genes and environment in prenatal and early postnatal periods appears to be critical for the onset of multiple diseases in adulthood. Important factors influencing this interaction include genetic predisposition, regulation of gene expression, and changes in microbiota. Premature birth and intrauterine growth restriction (IUGR) are other important factors considered by the DOHaD hypothesis. Preterm birth is associated with impaired or arrested structural or functional development of key organs/systems, making preterm infants vulnerable to cardiovascular, respiratory, and chronic renal diseases during adulthood. Growth restriction, defined as impaired fetal growth compared to expected biological potential in utero, is an additional negative factor increasing the risk of subsequent diseases. Environmental factors implicated in the developmental programming of diseases include exposure to pollution, stress, drugs, toxic agents, nutrition, and exercise. The DOHaD may explain numerous conditions, including cardiovascular, metabolic, respiratory, neuropsychiatric, and renal diseases. Potential antenatal and postnatal preventive measures, interventions, and future directions are discussed.

## 1. Introduction

The concept that early life events predict adult health and disease was initially proposed in 1986, when Barker et al. showed that adults who had low birth weight (<2.5 kg) were at higher risk of cardiovascular disease [1]. Since then, the concept of developmental programming has been extended to other organs and systems. The “developmental origins of health and disease” (DOHaD) hypothesis refers to the influence of early developmental exposures and fetal growth on the risk of chronic diseases in later periods. Cell differentiation and tissue formation occur in fetal and early postnatal life under the influence of several factors. It is increasingly recognized that perinatal period is of paramount importance for the development and the prevention of subsequent diseases. Neonatologists and pediatricians have an important “window of opportunity” to prevent and cure several diseases and, importantly, promote adult health.

In this narrative review, we will propose examples of diseases and discuss potential preventive measures with potential long-term impacts.

## 2. Developmental Programming of Diseases and Relative Mechanisms

Critical perinatal factors influencing organogenesis and predisposition to disease include genetic factors, interaction between genes and environment, duration of gestation, and maternal–fetal interactions.

The interaction between genes and the environment in prenatal and early postnatal periods appears to be critical for the onset of diseases in adulthood and has the potential to be modified by interventions. Important factors influencing this interaction include regulation of gene expression and changes in microbiota (individual microorganisms) and microbiome (their collective genomes) [2]. Across perinatal periods, multiple epigenetic mechanisms regulate gene expression without exerting modifications in the DNA sequence: examples are DNA methylation, histone modifications, chromatin remodeling, and transmission of small non-coding RNA. Maternal and paternal contributions to inheritance by means of epigenetic changes in response to nutritional factors and exposure to environmental agents (i.e., drugs, radiations) have recently been reported [3].

Premature birth and intrauterine growth restriction ( IUGR) are other important factors considered by the the DOHaD hypothesis. Preterm birth is associated with impaired or arrested structural or functional development of key organs/systems, making preterm infants vulnerable to several diseases at adulthood [4].

Another implication of preterm birth is the lack of hormonal supply with steroid hormones (estradiol and progesterone), which is typically observed among term infants. Both hormones increase up to 100-fold during pregnancy in the mother and the fetus. After preterm birth, these hormones drop dramatically in the mother and the newborn within hours. This is a physiological event at term, but the very preterm infant is disrupted from this huge hormonal supply at a much earlier developmental stage. Preliminary clinical data showed that the replacement of estradiol and progesterone in very preterm infants may improve lung development and neurological outcome [5,6].

Growth restriction, defined as impaired fetal growth compared to expected biological potential in utero, is an additional negative factor increasing the risk of subsequent diseases [7]. Fetal growth is determined by a complex interplay between genetic factors, nutrient and oxygen availability from the placenta, environmental factors, and endocrine modulation of these interactions [7].

True IUGR, compared to constitutional smallness, is a pathological condition in which the placenta fails to deliver an adequate supply of oxygen and nutrients to the developing fetus [8]. Differential expression of growth factors, proteins, and mRNA in placentas of women who delivered growth-restricted fetuses have been reported, suggesting the activation of compensatory mechanisms aimed at maximizing fetal growth [9].

Infants with IUGR, compared appropriately grown gestational age infants, have a significantly higher risk of mortality and neonatal complications with long-term consequences [10,11,12]. The etiology of these complications is due to fetal chronic hypoxia and nutrient deprivation due to placental dysfunction, with impaired fetal hemodynamic adaptations and subsequently altered organ structure and function [13]. For the prevention of IUGR, there is evidence that aspirin modestly reduces small-for-gestational-age (SGA) pregnancy in women at high risk and that a dose of ≥100 mg should be recommended and start at or before 16 weeks of gestation [14]. However, the optimal strategy to identify women who may benefit from prophylactic aspirin still has to be determined.

Changes in microbial population and their interactions with genes and the environment in different organs (i.e., intestine, lungs) have been linked to the development of several diseases, including metabolic syndrome, cardiovascular diseases, and respiratory and psychiatric disorders [2,15].

Suboptimal nutrition and extrauterine growth restriction also increase the risk of complications of prematurity [16]. However, excessive catch-up growth may have negative effects on lifespan [17]. Epigenetic alterations, altered insulin sensitivity, and antioxidant capacity resulting in tissue remodeling and telomere shortening seem to play a significant role in these complications [18,19].

Environmental factors implicated in the developmental programming of diseases include exposure to pollution, stress, drugs, toxic agents, exercise, and nutrients [20,21,22].

## 3. Cardiovascular, Renal and Metabolic Disease

Preterm birth and IUGR can result in structural changes of the cardiovascular system, such as the development of short sarcomeres in cardiomyocytes and vascular remodeling (muscularization), resulting in increased arterial stiffness. IUGR and preterm birth are independent risk factors for the development of subsequent cardiovascular disease and **hypertension** [23,24,25]. The cause of hypertension is likely multifactorial (i.e., reduced nephron number, increased arterial stiffness) and affected by both prenatal and postnatal events. Protein malnutrition, pharmacologic exposures, and hypoxia are important causes of a reduction in glomeruli, and reduced nephron number due to altered programming has been considered an important factor associated with elevated blood pressure [4,26].

According to a recent study, young adults born preterm have evidence of greater diffuse myocardial fibrosis in the left ventriculum that relates to the degree of prematurity, resulting in impaired diastolic function [27]. Greater diffuse myocardial fibrosis may underlie part of the increased cardiovascular risk in this population, including heart failure, ischemic heart disease, and early cardiovascular-related mortality.

Infants born afterIUGR have increased vascular stiffness and increased intima-media thickness, resulting in decreased vascular compliance and impaired endothelial-dependent vasodilation. These vascular alterations may increase myocardial workload and contribute to the development of hypertension in adulthood [28,29,30].

Bassareo et al. suggested that central aortic elasticity in former extremely preterm infants is impaired when compared with that of term-born controls, particularly in the context of intrauterine growth restriction [31]. Animal models of intrauterine growth restriction demonstrated an altered elastin to collagen ratio (with less elastin and increased collagen engagement, which is 100–1000 times stiffer than elastin), which in turn led to increased arterial stiffness [32]. Elastin slowly accumulates during late gestation and early neonatal period, and slowly involves during aging, with resulting progressive collagen increase [33]. Furthermore, previous reports highlighted that subjects who were born preterm may develop an early peripheral arterial dysfunction—that is, the first manifestation of atherosclerosis, preceding structural changes in the vascular wall [34]. Johansson et al. showed an increase of blood pressure in adults born preterm, adjusted for birth weight and current body mass index [35]. Hypertension has both an early onset, with up to 70% of preterm infants having elevated systolic blood pressure in infancy, and a prolonged duration, with hypertension remaining a significant concern into adulthood, particularly in the presence of adult obesity [36,37].

The postnatal environment plays an important role in reducing or enhancing the likelihood of disease expression: proposed postnatal factors include nutrient availability and stress [38]. In their longitudinal study, Barker et al. reported that children who developed hypertension later in life were characterized by slow fetal growth, followed by rapid compensatory growth in childhood [39]. Neonatal growth acceleration increases the risk of obesity-related hypertension [40], whereas continued growth failure increases the risk of hypertension beyond the effect of IUGR alone [41,42]. 

In a meta-analysis by Horta et al., breastfeeding, regardless of IUGR status, decreased the likelihood of developing major cardiovascular risk factors, including type 2 diabetes and obesity; however, no association was observed with blood pressure [43]. Lindberg et al. hypothesized that the association between low birth weight and increased risk of hypertension in adulthood may be modifiable with micronutrient interventions in infancy such as iron supplements, highlighting the need for ongoing nutritional assessment [44]. However, the role of perinatal micronutrient and iron deficiencies in relation to blood pressure level in adulthood is still under investigation.

Prematurity is also a major risk factor also for **obesity**, and the risk increases with decreasing gestational age [45]. Among others, the postnatal period is characterized by the fastest growth. It represents a critical window of tissue and organ development wherein several regulatory mechanisms continue to develop after birth. Variations in this process may have long-lasting effects on health. The association between weight gain in infancy and obesity in childhood, adolescence, and adulthood has been widely recognized [46]. Abdominal adipose tissue, an endocrine organ, secretes adipocytokines and vasoactive substances that can influence the risk of developing metabolic traits [47].

In addition to family history and unhealthy lifestyle factors, early life exposures have been identified as potential risk factors for the development of **diabetes** later in life. Preterm and early term birth were associated with up to 1.5-fold increased risk of type 1 and type 2 diabetes from childhood into early to mid-adulthood in a large population-based cohort [48]. These findings may have multiple underlying mechanisms that involve pancreatic beta cell function and insulin resistance. Preterm birth interrupts the development of pancreatic beta cells, which are formed predominantly in the third trimester of pregnancy, and might permanently reduce their number or function [49]. Preterm birth also alters immune function including T cell response, which may potentially mediate its association with type 1 diabetes, consistent with its autoimmune etiology [50]. Other contributing factors may include exposure to antenatal corticosteroids and rapid catch-up growth in infancy, leading to visceral adiposity and insulin resistance [51].

Most studies show a 30–40% reduction in insulin sensitivity in children and young adults born very preterm (<32 weeks’ gestation) in comparison with those born at term [52]. Another study showed that adults born even moderately preterm (32–36 weeks’ gestation) have an isolated reduction in insulin sensitivity but normal β-cell function [53].

**Stroke** is one of the most common causes of disability among adults worldwide. Previous studies have indicated that low birth weight is associated with an increased risk of adult stroke in men and that birth weight is inversely associated with the risk of stroke in women [54,55]. The risk of both ischemic and hemorrhagic stroke is associated with preterm birth [56]. Some studies have demonstrated increasing trends in the incidence of low birth weight and ischemic stroke among young adults also in middle- and low-income countries [57], making the identification of new risk factors and preventive measures a research priority.

In humans 60% of the nephrons develop during the third trimester of gestation, mostly between 28 and 34 weeks of gestation. The final endowment of nephrons is both dependent on gestational age at birth and intrauterine environment. The principal factor, among others, which determines nephron number is birth weight [58]. An event occurring during the early stage of nephrogenesis can have dramatic effects on the final nephron number. However, the number of nephrons can be ‘reprogrammed’ through various interventions (including nutritional interventions) applied during pregnancies at risk [59].

**Chronic kidney disease (CKD)** is defined as the reduction of reduced glomerular filtration rate (GFR) up to end-stage renal disease (ESRD), proteinuria, or both. Prevalence of ESRD is increasing worldwide. Reduced nephron endowment has been proposed as playing a determinant role in the pathogenesis of CKD [26]. Reduced nephron number is responsible for an adaptive glomerular hyperfiltration, resulting in renal hypertrophy and glomerular capillary enlargement. The consecutive glomerular hypertension may lead over time to renal injury, proteinuria, impaired GFR, and systemic hypertension [60]. Concomitant salt retention, increased peripheral vascular resistance and cardiac changes may lead to glomerular sclerosis, impaired GFR, and systemic hypertension. Eventually, inflammation, upregulation of the renin angiotensin system, and the production of nitric oxide and reactive species worsen renal injury [61]. Low birth weight and intrauterine growth restriction are both associated with a decreased nephron number, the latter condition reducing it by an average of 30–35%, whereas the effects of preterm birth are still unknown [62]. In preterm infants, nephrogenesis is expected to continue in a potentially unfavorable environment.

While rapid postnatal growth and/or overfeeding enhances the “vulnerability state” acquired in utero and accelerates the development of adult diseases (“mismatch hypothesis”), slow postnatal growth and breastfeeding in particular (possibly through reduced protein and sodium intakes) tend to prevent such diseases [63]. Nephron endowment may result from a complex process which integrates the interaction of the fetal environment (or postnatal environment in preterm infants) and the genetic background.

## 4. Respiratory Disease

Conditions such as prematurity and its complications, fetal growth restriction, and inflammation have been associated with long-term pulmonary morbidity, including **asthma**, in up to 75% of infants born below 30 weeks of gestation [64,65]. Premature delivery results in loss of the normal structural complexity of the lung and greater susceptibility to subsequent injury from infection or environmental factors such as smoking. Genetic susceptibility factors also play a role in reduced immunologic regulation needed for normal lung development and function [66]. Proposed mechanisms by which preterm birth may affect subsequent risk of asthma include genetic, perinatal, and environmental factors.

Early-life inflammatory insults, as in neonatal respiratory distress and bronchopulmonary dysplasia (BPD), may hamper the development of properly organized pulmonary interstitium, with consequences for acinar structure and function, peribronchial airway support, and elastic recoil pressures [67]. Early onset **chronic obstructive pulmonary disease** (COPD) has been observed in subsets of extremely preterm-born adults, as lung function will commence its normal age-related decline from subnormal levels, possibly at steeper trajectories [68]. It has been hypothesized that young adults born preterm, having failed to reach optimal peak lung function, will decline during adulthood with a steeper trajectory than those born at term, and that external factors including pollution, infection, and smoking could have a further detrimental effect on this decline [69]. Structural changes of the lungs following IUGR and inflammation (impaired alveolar and vascular development, muscularization of lung vessels, endothelial dysfunction) have been related to development of **BPD** and pulmonary hypertension [70]. BPD has been associated with significant pulmonary morbidity beyond the neonatal period, including the use of bronchodilators up to two years of age, frequent diagnosis of asthma later in childhood, and persistence of abnormal baseline spirometry at 11 years of age compared with full-term controls [71]. Patients with BPD are also more likely to be hospitalized after discharge from the neonatal intensive care unit and use outpatient services more frequently than premature patients without BPD [72,73]. However, according to other studies, premature infants without BPD are also at risk of developing pulmonary morbidity beyond the neonatal period as compared with term infants. An equal incidence of wheezing-related illnesses among patients born prematurely regardless of the presence of BPD was reported [64]. In a study of 25-year-old adults born extremely preterm in the early 1980s, exercise capacity was 10% lower than in a control group born at term (but still within a range considered normal) and was positively associated with self-reported physical activity and unrelated to neonatal factors and current airway obstruction [74].

Difficulties in this research area, characterized by a long time span between birth and the occurrence of complications, include the rapid evolution of obstetric and neonatal care (i.e., improved survival, implementation of preventive strategies such as prenatal steroids and caffeine, maternal metabolic control, newborn practices among others) that can have an important influence on long-term outcomes.

## 5. Neuropsychiatric Conditions

Increased risk of impaired neurodevelopment and psychological dysfunctions in the first four decades of life were reported in preterm infants, in small for gestational age (SGA) infants compared to appropriate for gestational age (AGA) infants and in pregnancies complicated by maternal diabetes [75,76,77]. Underlying mechanisms could be reduced brain volume, organizational differences, oxidative stress, and hypoxia of the fetus. In a Swedish cohort study, individuals who were born preterm were more likely to be prescribed psychotropic medications during young adulthood than individuals who were born full term [78]. Moreover, chronic diseases (i.e., Alzheimer’s disease, schizophrenia) may be associated with epigenetic factors, nutritional deficiency, and exposure to toxic agents occurring during gestation [79].

## 6. Potential Preventive Measures, Interventions and Future Directions

Several preventive measures can be identified and considered to promote long-term health. Examples of useful **antenatal** measures are: improved identification of subjects with increased risk of complications (i.e., earlier/more frequent ecographic growth assessment), dietary modifications during pregnancy to ensure normalization of body weight, zinc and iron levels, glycemia and blood pressure control, lifestyle measures (i.e., avoidance of alcohol and tobacco, maximization of maternal education), reduced stress and exposure to pollution), and management of chronic diseases. Some of these measures are currently being evaluated in the context of clinical studies [80,81,82,83,84,85,86,87,88].

The prevention of preterm birth and enhanced maturation (optimal antenatal steroid administration) is of paramount importance. Global policies to enhance health, particularly in low-income countries have been advocated [89]. Specific dietary interventions, including the supplementation of folic acid, zinc, long-chain polyunsaturated fatty acids, and vitamin D, which are possibly associated with favorable epigenetic changes, are under assessment [15].

Finally, the administration of drugs during high-risk pregnancies (i.e., when IUGR is demonstrated) is another potential measure: sildenafil has been investigated but increased fetal death in a clinical trial has led to discontinuation of the study [90]; vascular endothelial growth factor is currently under investigation to promote angiogenesis [91], insulin-like growth factor 1 (IGF-1), antioxidants and melatonin have been tested in preclinical studies [92,93,94]. The identification of the optimal timing of delivery in pathologic conditions (such as IUGR) is another important aspect, and studies are underway in this regard [95].

**Postnatal** interventions in the early phases of life include promotion of breastfeeding, optimization of nutrition and growth (potentially with administration of hormones/growth factors such as IGF-1 analogues, cautious use and therapeutic drug monitoring of toxic drugs (i.e., nephrotoxic antibiotics, systemic steroids with potential heart and brain toxicity), adequate follow-up of patients at high risk, appropriate resource allocation [89]. The change of maternal and offspring microbiota by dietary modifications (i.e., dietary supplementation with docosahexaenoic acid and arachidonic acid to improve neurodevelopmental outcomes) [83], pre-probiotics, and possibly other factors is a potential intervention needing further studies.

Novel drugs under investigation include lactoferrin and stem cell administration [96,97].

Knowledge translation, the process of putting knowledge into action, is of paramount importance to ensure the use of research findings in decision-making [98]. In fact, the prevention of preterm birth, IUGR, and their long-term complications, as here discussed, is highly relevant for individual and public health. One approach could be to analyze and compare strengths and characteristics of different health systems to inform clinical decision-making, research, and healthcare policy, as recently performed by Japanese and Canadian researchers regarding the prevention and management of preterm birth [99].

In conclusion, developmental programming is emerging as a new concept for the explanation of several diseases in children and adults. The characterization of underlying mechanisms and the identification of preventive measures and treatment are of great importance in order to promote health and prevent the development of several chronic diseases.
